# Supplementary figures and images for: Host tissue proteomics reveal insights into the molecular basis of Schistosoma haematobium-induced bladder pathology
Source: PLoS Negl Trop Dis. 2022 Feb 15;16(2):e0010176. doi: 10.1371/journal.pntd.0010176 (PMC8846513; doi:10.1371/journal.pntd.0010176)

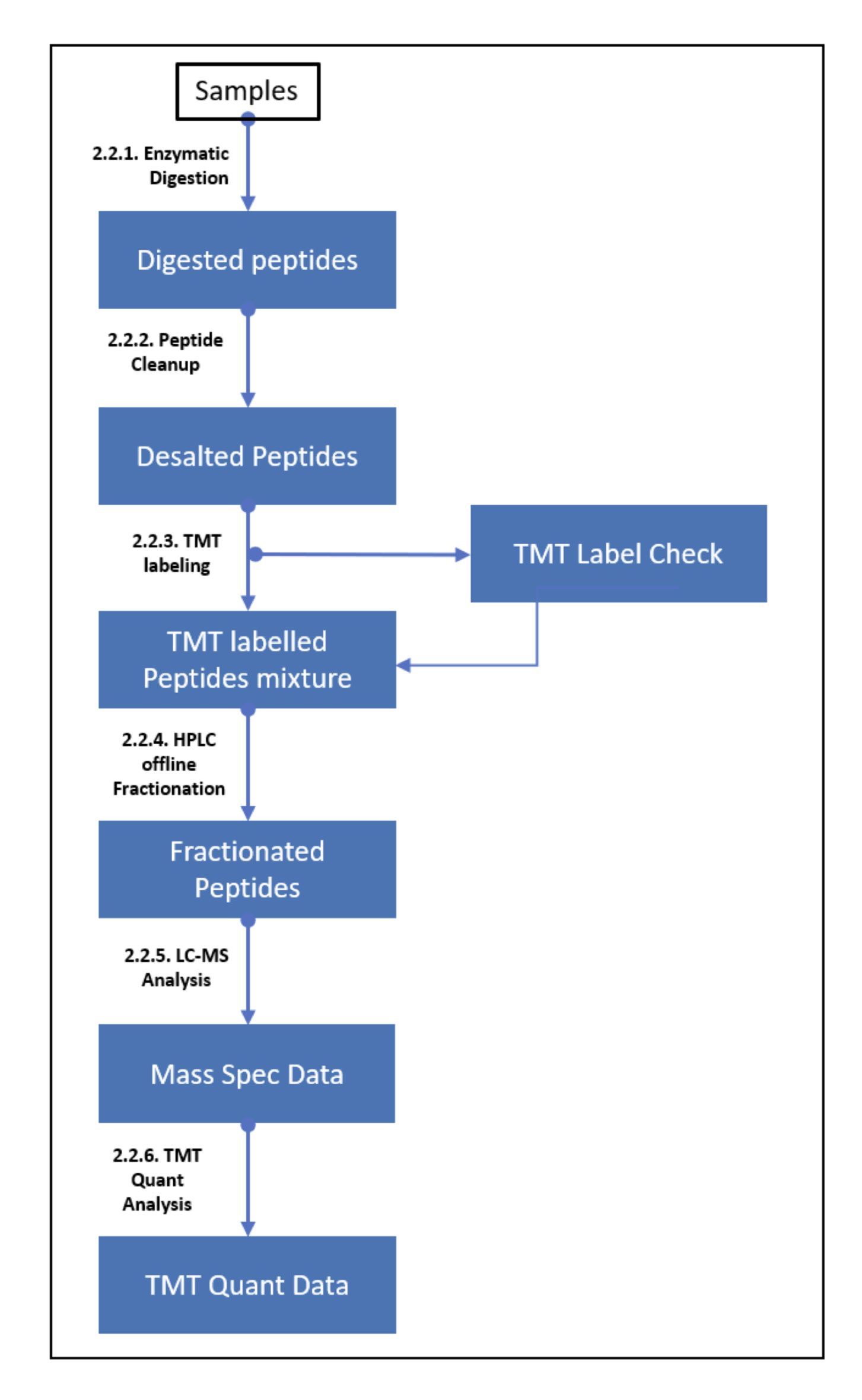

Supplement: S1 Fig — (TIFF) [file pntd.0010176.s001.tiff]

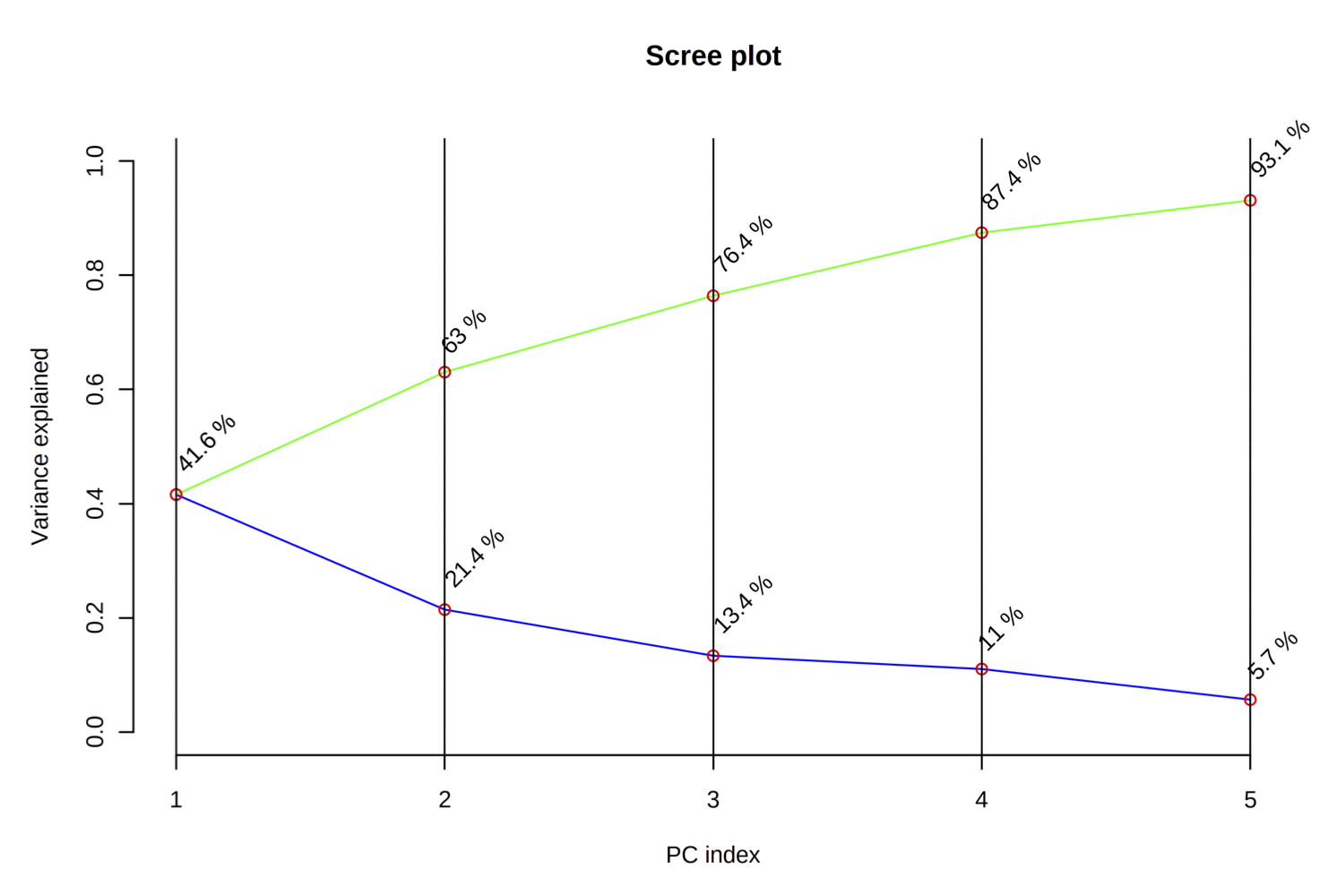

Supplement: S2 Fig — The green line on top shows the accumulated variance explained; the blue line underneath shows the variance explained by individual principal components. (TIFF) [file pntd.0010176.s002.tiff]

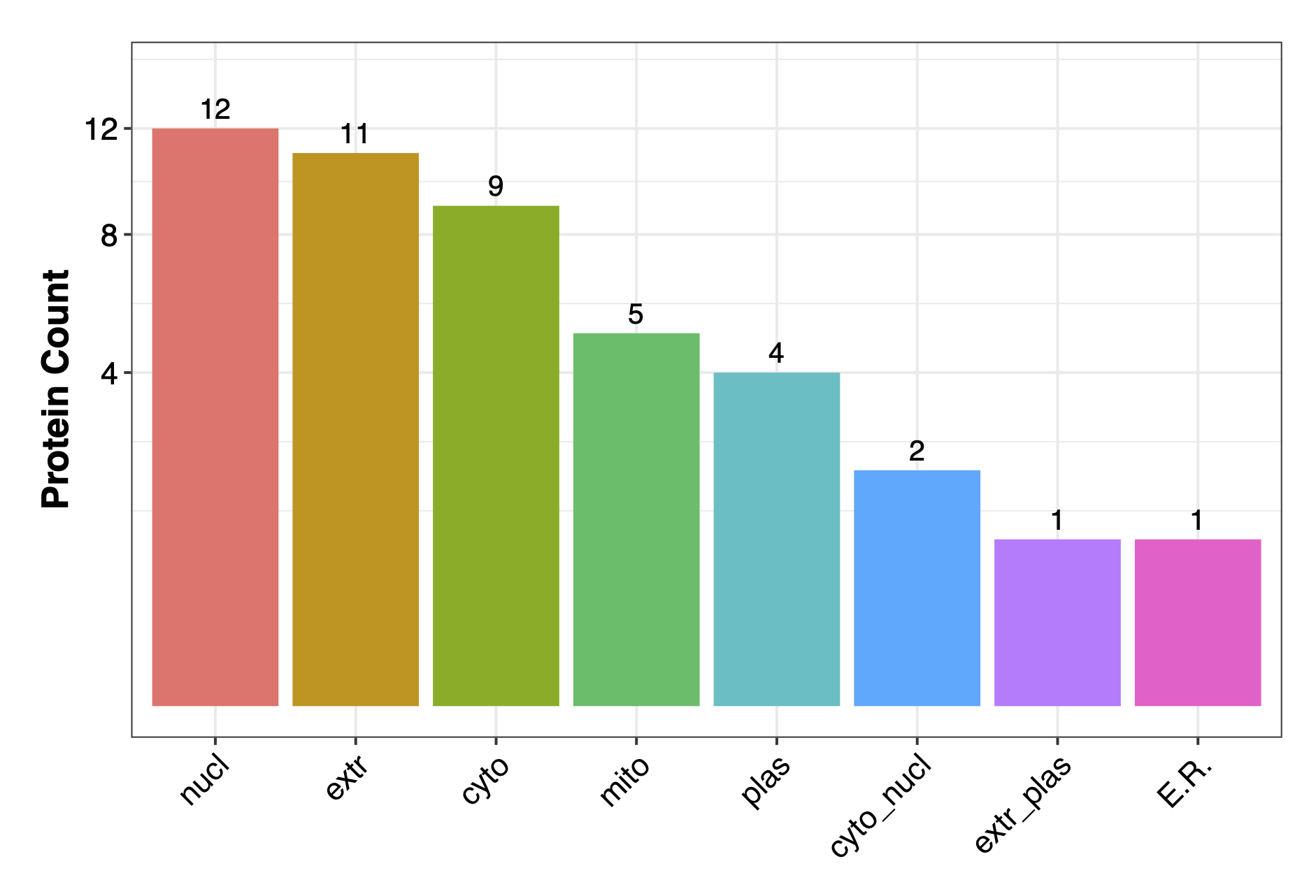

Supplement: S3 Fig — E.R, endoplasmic reticulum; cyto, cytosol; cyto_nucl, cytosol or nucleus; extr, extracellular; extr_plas, extracellular or plasma membrane; mito, mitochondrial; nucl, nucleus; plas, plasma membrane. (TIFF) [file pntd.0010176.s003.tiff]
