# Supplementary material for: Host tissue proteomics reveal insights into the molecular basis of Schistosoma haematobium-induced bladder pathology
Source: PLoS Negl Trop Dis. 2022 Feb 15;16(2):e0010176. doi: 10.1371/journal.pntd.0010176 (PMC8846513; doi:10.1371/journal.pntd.0010176)
Supplement: S1 Table — (PDF) [file pntd.0010176.s004.pdf]

S1 Table. Samples and their corresponding TMT tag channels.

Sample #	Sample Name	Tag Channel
1	T1	126
2	T2	127N
3	T3	127C
4	T4	128N
5	T5	128C
6	Sh1	129N
7	Sh2	129C
8	Sh3	130N
9	Sh4	130C
10	Sh5	131
