# Supplementary material for: Host tissue proteomics reveal insights into the molecular basis of Schistosoma haematobium-induced bladder pathology
Source: PLoS Negl Trop Dis. 2022 Feb 15;16(2):e0010176. doi: 10.1371/journal.pntd.0010176 (PMC8846513; doi:10.1371/journal.pntd.0010176)
Supplement: S2 Table — (PDF) [file pntd.0010176.s005.pdf]

S2 Table. High pH reverse phase HPLC fractionation gradient information

Time [min]	Flow [ml/min]	%B
0.00	0.500	2.0
1.00	0.500	6.0
12.00	0.500	20.0
30.00	0.500	28.0
50.00	0.500	65.0
53.00	0.500	98.0
57.00	0.500	98.0
59.00	0.500	2.0
60.00	0.500	2.0

%B, Mobile phase B (Acetonitrile (OptimaTM, LC/MS grade, Fisher ChemicalTM) with 20mM Formic Acetate, pH 9.3).
