# Supplementary material for: Host tissue proteomics reveal insights into the molecular basis of Schistosoma haematobium-induced bladder pathology
Source: PLoS Negl Trop Dis. 2022 Feb 15;16(2):e0010176. doi: 10.1371/journal.pntd.0010176 (PMC8846513; doi:10.1371/journal.pntd.0010176)
Supplement: S3 Table — (PDF) [file pntd.0010176.s006.pdf]

S3 Table. nanoLC-MS gradient information

Time[min]	Flow[µl/min]	%B
0.00	0.300	2.0
3.00	0.300	2.0
3.10	0.300	2.0
8.00	0.300	4.0
108.00	0.300	35.0
128.00	0.300	65.0
129.00	0.300	100.0
133.00	0.300	100.0
134.00	0.300	2.0
140.00	0.300	2.0
150.00	0.300	2.0

%B, Mobile phase B (Acetonitrile (OptimaTM, LC/MS grade, Fisher ChemicalTM) with 20mM Formic Acetate, pH 9.3.
