# Supplementary material for: Host tissue proteomics reveal insights into the molecular basis of Schistosoma haematobium-induced bladder pathology
Source: PLoS Negl Trop Dis. 2022 Feb 15;16(2):e0010176. doi: 10.1371/journal.pntd.0010176 (PMC8846513; doi:10.1371/journal.pntd.0010176)
Supplement: S3 File — (PDF) [file pntd.0010176.s019.pdf]

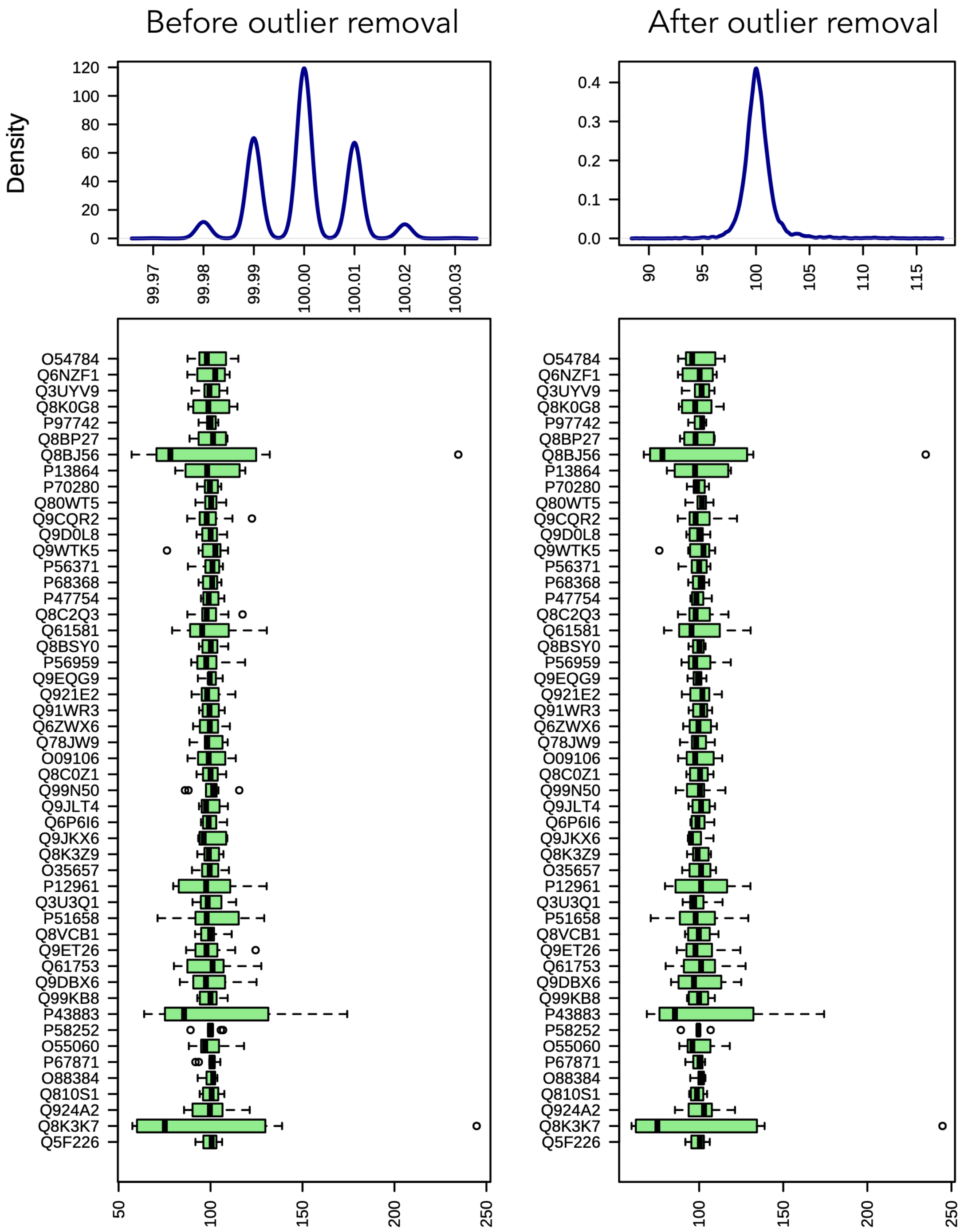

Box plots and kernel density plots before (n=10; Cases=5, Controls=5) and after (n=8; Cases=4, Controls=4) removal of outliers from dataset. The boxplots show a maximum of 50 proteins due to space limit. The density plots are based on all samples before and after outlier removal.
